# Pharmacological targeting of KDM6A and KDM6B, as a novel therapeutic strategy for treating craniosynostosis in Saethre-Chotzen syndrome

**DOI:** 10.1186/s13287-020-02051-5

**Published:** 2020-12-09

**Authors:** Clara Pribadi, Esther Camp, Dimitrios Cakouros, Peter Anderson, Carlotta Glackin, Stan Gronthos

**Affiliations:** 1 Mesenchymal Stem Cell Laboratory, Adelaide Medical School, Faculty of Health and Medical Sciences, University of Adelaide, Adelaide, South Australia Australia; 2 Precision Medicine Theme, South Australian Health and Medical Research Institute, Adelaide, South Australia Australia; 3 Adelaide Craniofacial Unit, Women and Children Hospital, North Adelaide, South Australia Australia; 4 Molecular Medicine and Neurosciences, City of Hope National Medical Center and Beckman Research Institute, Duarte, CA USA

**Keywords:** Epigenetics, KDM6A, KDM6B, Calvarial cells, Osteogenesis, Coronal sutures, TWIST-1, *Twist-1*^del/+^ mice, Saethre-Chotzen syndrome, Craniosynostosis

## Abstract

**Background:**

During development, excessive osteogenic differentiation of mesenchymal progenitor cells (MPC) within the cranial sutures can lead to premature suture fusion or craniosynostosis, leading to craniofacial and cognitive issues. Saethre-Chotzen syndrome (SCS) is a common form of craniosynostosis, caused by *TWIST-1* gene mutations. Currently, the only treatment option for craniosynostosis involves multiple invasive cranial surgeries, which can lead to serious complications.

**Methods:**

The present study utilized *Twist-1* haploinsufficient (*Twist-1*^del/+^) mice as SCS mouse model to investigate the inhibition of Kdm6a and Kdm6b activity using the pharmacological inhibitor, GSK-J4, on calvarial cell osteogenic potential.

**Results:**

This study showed that the histone methyltransferase *EZH2*, an osteogenesis inhibitor, is downregulated in calvarial cells derived from *Twist-1*^del/+^ mice, whereas the counter histone demethylases, *Kdm6a* and *Kdm6b*, known promoters of osteogenesis, were upregulated. In vitro studies confirmed that siRNA-mediated inhibition of *Kdm6a* and *Kdm6b* expression suppressed osteogenic differentiation of *Twist-1*^del/+^ calvarial cells. Moreover, pharmacological targeting of Kdm6a and Kdm6b activity, with the inhibitor, GSK-J4, caused a dose-dependent suppression of osteogenic differentiation by *Twist-1*^del/+^ calvarial cells in vitro and reduced mineralized bone formation in *Twist-1*^del/+^ calvarial explant cultures. Chromatin immunoprecipitation and Western blot analyses found that GSK-J4 treatment elevated the levels of the Kdm6a and Kdm6b epigenetic target, the repressive mark of tri-methylated lysine 27 on histone 3, on osteogenic genes leading to repression of *Runx2* and *Alkaline Phosphatase* expression. Pre-clinical in vivo studies showed that local administration of GSK-J4 to the calvaria of *Twist-1*^del/+^ mice prevented premature suture fusion and kept the sutures open up to postnatal day 20.

**Conclusion:**

The inhibition of Kdm6a and Kdm6b activity by GSK-J4 could be used as a potential non-invasive therapeutic strategy for preventing craniosynostosis in children with SCS.

**Graphical abstract:**

Pharmacological targeting of Kdm6a/b activity can alleviate craniosynostosis in Saethre-Chotzen syndrome. Aberrant osteogenesis by Twist-1 mutant cranial suture mesenchymal progenitor cells occurs via deregulation of epigenetic modifiers Ezh2 and Kdm6a/Kdm6b. Suppression of Kdm6a- and Kdm6b-mediated osteogenesis with GSK-J4 inhibitor can prevent prefusion of cranial sutures. 
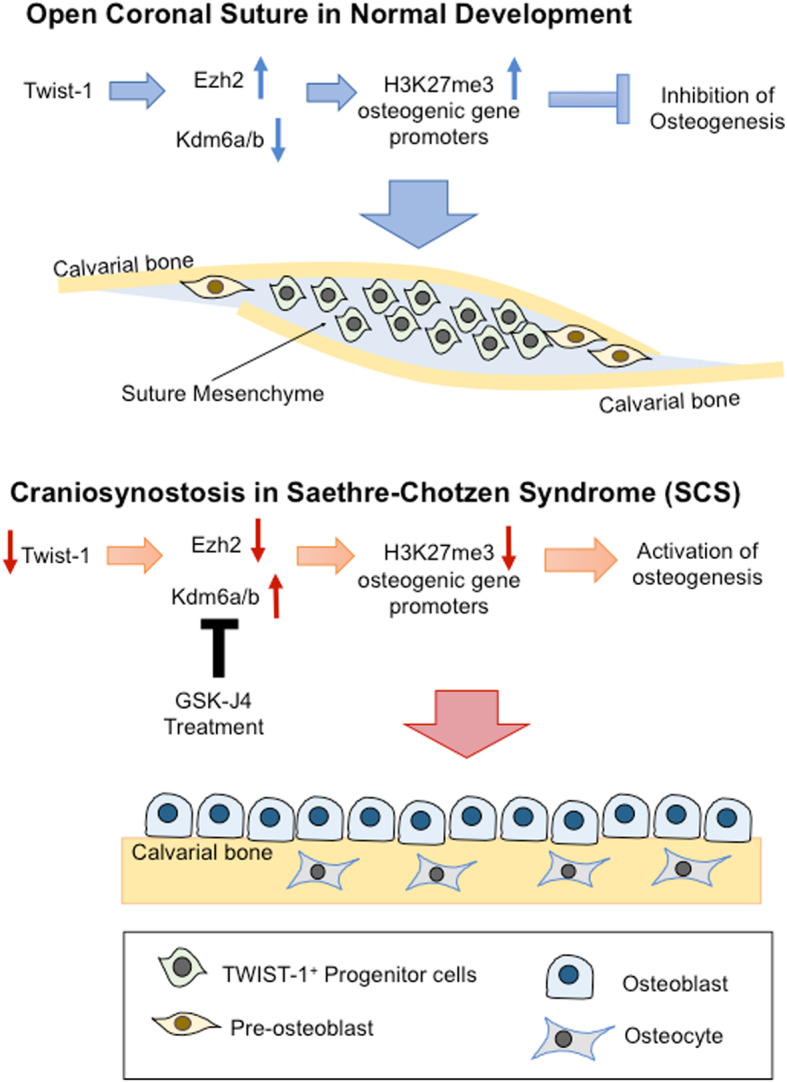

## Background

Calvarial sutures are comprised of active mesenchyme forming the osteogenic fronts at the edges of the flat calvarial bones [1, 2]. Within the suture mesenchyme, a reservoir of TWIST-1^+^/Gli-1^+^ mesenchymal progenitor cells (MPC) exists which can either remain undifferentiated or differentiate to mature bone forming osteoblasts [3–7]. During embryonic and postnatal development, the sutures remain open up to adulthood, providing flexibility to the calvaria and allowing the cranium to accommodate for the growing brain [8–11]. However, dysregulation of MPC differentiation within these sutures results in excessive intramembranous ossification and premature fusion of the suture space or called craniosynostosis.

Craniosynostosis occurs in 1 in 2500 live births and can result in an unusual head shape, facial asymmetry, and most importantly, pre-fusion of the cranial sutures, causing increased pressure on the developing brain leading to neurological deficits [12, 13]. Currently, the only treatment for craniosynostosis is invasive cranial surgery, mainly involving the removal of the affected sutures and remodeling of the skull [14, 15]. These procedures could negatively impact the quality of life of children with craniosynostosis, leading to serious complications and enforcing the need for invasive surgical procedures [16–18]. The most prevalent syndromic craniosynostosis, Saethre-Chotzen syndrome (SCS), involves unilateral and bilateral coronal synostosis, facial asymmetry, occasional cleft palate, droopy eyelid, and mild limb deformities such as the shortened and united fingers and toes [19, 20]. SCS is induced by the deletion or non-sense mutations resulting in the loss of function or haploinsufficiency of the *TWIST-1* gene [21]. There are more than 100 different *TWIST-1* gene mutations related to SCS, resulting in a range of phenotypes from a simple unilateral coronal synostosis to a complex multiple suture synostosis [22]. Recently, it has been revealed that epigenetic mechanisms play a significant role in craniosynostosis where studies of genetically identical twins reported that one twin displayed craniosynostosis, whereas the other displayed normal skull development [23, 24]. These observations suggest that the development of craniosynostosis in only one identical twin is most likely due to epigenetic changes. However, until now, no study has thoroughly examined the role of epigenetics in SCS.

TWIST-1, a basic helix-loop-helix transcription factor, has been shown to mediate skeletal and head tissue development [9, 25, 26]. Its expression in MPC within the calvarial sutures is essential in maintaining its stemness characteristics, such as proliferation activity, and negatively regulating osteogenic differentiation by directly inhibiting major osteogenic genes [9, 27–30]. Furthermore, previous studies found that *Twist-1* expression is required for correct establishment of the coronal sutures in mice [5, 31]. Haploinsufficiency of the *TWIST-1* gene in SCS-derived calvarial cells results in a decrease in proliferation and increased osteogenic differentiation, leading to premature suture fusion [28, 32, 33]. *TWIST-1* expression and function have been correlated with the epigenetic regulator Enhance of Zeste Homolog 2 (EZH2) in mediating SCS cranial bone cell growth and differentiation [32], where *Ezh2* knockdown in the mesenchymal lineage leads to craniosynostosis and other skeletal deformities [34]. EZH2 is a member of the Polycomb Repressive Complex 2 and acts as a methyltransferase which tri-methylates lysine-27 of the histone-3 tail (H3K27me3), to repress gene activation [35]. The counter demethylases, UTX (lysine demethylase 6A, KDM6A) and JMJD3 (lysine demethylase 6B, KDM6B), remove the tri-methylation mark on H3K27me3 to promote gene activation [36–39]. The enzymatic demethylase activity of these epigenetic modifiers is carried out by the Jumonji C catalytic domain, through a dioxygenase reaction that requires Fe (II) and α-ketoglutarate as co-substrates [40]. Previous studies have reported that KDM6A and KDM6B promote osteogenic differentiation of mesenchymal stem cells [41, 42], whereas EZH2 represses bone gene activation and mesenchymal stem cell osteogenic differentiation [41]. Additionally, loss-of-function mutation of KDM6A has been previously identified to be associated with a congenital skeletal tissue disorder, called Kabuki syndrome, with characteristics including malformed cranial bones [43–45]. Similarly, loss of KDM6B results in a severe delay of osteogenic differentiation in mice [46, 47] and lowered expression of *Runx2* and *Osterix*, as a result of increased levels of H3K27me3 [48]. These observations provide affirmation that both KDM6A and B play important roles in promoting osteogenic differentiation of MPC.

To further examine epigenetic changes in SCS, we utilized *Twist-1* heterozygous mutant mice (*Twist-1*^del/+^), which display craniofacial defects including unilateral or bilateral coronal synostosis and limb abnormalities similar to the characteristic abnormalities described for SCS human patients [49]. The present study investigated the expression levels and role of *Kdm6a* and *Kdm6b* in the osteogenic potential of calvarial cells and calvarial explants derived from *Twist-1*^del/+^ mice. Furthermore, we assessed a potential drug therapy approach to reverse aberrant osteogenic differentiation in the sutures of *Twist-1*^del/+^ mice using the small-molecule cell-permeable selective inhibitor, GSK-J4 targeting Kdm6a and Kdm6b activity in vivo.

## Methods

### Isolation of mouse calvarial cells

Mouse calvarial stromal cells were derived from the calvaria of 15-day-old *Twist-1*^del/+^ heterozygous mice and wildtype mice in accordance with South Australia Health and Medical Institute (SAHMRI) Animal Ethics Committee approval # SAM347. The mice were humanely killed by CO_2_ inhalation followed by cervical dislocation. The calvarial bones were retrieved then digested twice with Collagenase I (3 mg/mL) and DNAase I (50 U/mL) in PBS for 40 min each time. Bone chips were cultured under hypoxia condition (5% O_2_) with growth media, α-Modification of Eagle’s medium (αMEM) supplemented with 20% fetal calf serum (Batch F21701; CellSera, Rutherford, NSW, AUS), 1 mM sodium pyruvate, 2 mM L-glutamine, 50 U/mL penicillin, and 50 μg/mL streptomycin for up to 7 days. Cells were disassociated with Collagenase I (3 mg/mL) and Dispase (3 mg/mL) solution, cultured with a seeding density of 8 × 10^3^ cells/cm^2^ and grown until confluent.

### Osteogenic differentiation assays

Cells were cultured in osteogenic inductive media (αMEM supplemented with 10% fetal calf serum, 100 μg/mL L-ascorbate-2-phosphate, 10 mM β-glycerol phosphate, 2 mM L-glutamine, 1 mM sodium pyruvate, 10 mM HEPES buffer, 1 × 10^−8^M dexamethasone, 50 U/mL penicillin, and 50 μg/mL streptomycin) for 1 week or 2 weeks. GSK-J4 at 1 μM and 2 μM or 0.1% dimethyl sulfoxide (DMSO) in osteogenic-inductive media were refreshed every 24 h. Alkaline phosphatase staining was performed using Leukocyte Alkaline Phosphatase Kit (Cat# 86R-1KT, Sigma–Aldrich Inc., North Ryde, NSW, AU) following manufacturer’s protocols. The activity of alkaline phosphatase was quantitated in triplicate and normalized to total protein level per well using Alkaline Phosphatase Assay Kit (Cat# ab83369, Abcam Australia Pty Ltd., Melbourne, VIC, AU), following manufacturer’s instructions. Bone mineral deposits were stained with Alizarin red S (Cat# A5533, Sigma–Aldrich, Inc.), and extracellular calcium levels were measured in triplicate wells and normalized to DNA content per well as previously described [27].

### siRNA gene knockdown studies

Cells were seeded at 3 × 10^4^ cells per well in 24-well plate the day before siRNA transfections to achieve approximately 70% confluency. Sequence-specific siRNA against *Kdm6a* (s75838 and s75839) and *Kdm6b* (s103747 and s103746) or negative siRNA#1 control (Ambion/Life Technologies, Scoresby, VIC, AU) were transfected into the cells at concentration of 20 pmol in transfection medium (αMEM with 10% fetal calf serum) with Lipofectamine RNAiMAX reagent (Thermo-Fisher Scientific, Scoresby, VIC, AU) as previously described [41]. The incubation period for the transfection to achieve at least a 50% knockdown of transcript levels was 72 h before changing the media to osteogenic inductive media.

### GSK-J4 treatment

GSK-J4 (Cat# 12073, Cayman Chemical, Ann Arbor, MI, US) was reconstituted in DMSO and stored at − 80 °C. Cells were seeded at 4.2 × 10^4^ cells per well into 24-well plate. GSK-J4 at 0.1 μM, 0.25 μM, 0.5 μM, 1 μM, 2 μM, 5 μM, and 10 μM or DMSO (0.1%) only were added to the cells in the presence of either growth or osteogenic inductive media.

### Gene expression studies

Total RNA from cultured *Twist-1*^del/+^ calvarial cells was isolated using TRIzol reagent (Cat# 15596026, Invitrogen/Thermo Fisher Scientific, Waltham, MA, USA), according to manufacturer’s instructions. Synthesis of cDNA and real-time polymerase chain reaction (PCR) analysis were performed in triplicate as previously described [50]. Primer sets (GeneWorks Pty Ltd., Thebarton, SA, AU) used in this study were mouse *β-Actin* (Fwd: 5′-TTGCTG ACAGGATGCAGAAG-3′; Rev.: 5′-AAGGGTGTAAAACGGAGCTC-3′); mouse *Kdm6a* (Fwd: 5′-GGCTACTGGGGTGTTTTGAA-3′; Rev.: 5′-TCCAGGTCGCTGAATAAACC-3′); mouse *Kdm6b* (Fwd: 5′-CCCCCATTTCAGCTGACTAA-3′; Rev.: 5′-CTGGACCAAGGGGTGTGTT-3′); mouse *Ezh2* (Fwd: 5′-ACTGTCGGCACCGTCTGATG-3′; Rev.: 5′-TCCTGAGAAATAATCTCCCCACAG-3′); mouse *Twist-1* (Fwd: 5′-CAGCGGGTCATGGCTAAC-3′; Rev.: 5′-TCCTGAGAAATAATCTCCCCACAG-3′); mouse *Alkaline Phosphatase* (Fwd: 5′-GCCTTACCAACTCTTTTGTGC-3′; Rev.: 5′-GGCTACATTGGTGTTGAGCTT-3′); mouse *Runx2* (Fwd: 5′-CCTCTGACTTCTGCCTCTGG-3′; Rev.: 5′-TATGGAGTGCTGCTGGTCTG-3′).

### Cell proliferation assay

Cells were cultured at 9 × 10^3^ cells/well in 96-well plates in the presence of DMSO (0.1%) or a range of GSK-J4 concentrations (0.1 μM, 0.25 μM, 0.5 μM, 1 μM, 2 μM, 5 μM, and 10 μM) in growth inductive media (αMEM supplemented with 20% fetal calf serum, pyruvate, L-glutamine, P/S) for 7 days. The rate of cell proliferation was measured using cell proliferation ELISA, bromodeoxyuridine (BrdU) colorimetric kit (Cat# 11647229001, Roche Products Pty Limited, Sydney, NSW, AU), following manufacturer’s directions. Absorbance was read at 450 nm on an iMark microplate reader (Bio-Rad Laboratories, Hercules, CA, USA).

### Cell viability assay

Cells were seeded at 2.6 × 10^5^ cells/well into 6-well plates in growth inductive media and in the presence of 0.1% DMSO or GSK-J4 concentration range (0.1 μM–10 μM) for 7 days. The rate of apoptosis was measured using Annexin V and 7AAD staining procedure. For positive controls, apoptosis and necrosis were induced by adding 100% DMSO overnight and 70% Ethanol, respectively. Prior to reading, 5 μL of Annexin V-488 (Cat# A13202, Invitrogen/Thermo Fisher Scientific) and 20 μL of 7-amino-actinomycin (7AAD; Cat# A1310, Invitrogen/Thermo Fisher Scientific) were added to ~ 1 × 10^6^ cells as previously described [33]. Samples were analyzed immediately on LSRForessa X20 Analyzer (BD Biosciences, North Ryde, NSW, Australia).

### Calvaria organ explant cultures

Whole calvaria organ explants isolated from 4-day-old *Twist-1*^del/+^ mice were placed onto mesh structures in the presence of BjGb media (Fitton-Jackson Modification with L-Glutamine; Cat# B1091, US Biological, MA, USA) with rhBMP2 (50 ng/mL, Cat# PHC7145, Thermo Fisher Scientific) and GSK-J4 at 1 μM or GSK-J4 at 2 μM or vehicle control (0.1% DMSO) for 10 days as previously described [51, 52]. Calvaria explants were then fixed in 10% formalin for 6 h, decalcified overnight with 14% EDTA (pH 7.2) and embedded in paraffin. Sections (7 μm) were stained with Masson’s trichrome staining. The formation of the mineralized bone shown in blue staining relative to the length of calvaria bone specimen was measured using OsteoMeasure XP Advanced Bone Histomorphometry ver.1.0.3.1 software (OsteoMetrics, Inc., Decatur, GA, US) on an Olympus BX53Microscope (Olympus, Notting Hill, VIC, Australia).

### In vivo administration of GSK-J4 to calvaria of *Twist-1*^*del/+*^ mice

Two 3 mm^3^ CollaCote sponges (Cat# 0101, Integra Life Sciences Services, Saint Priest, FRA) soaked in 0.1% DMSO as vehicle control or in GSK-J4 in the concentration of 2 μM was placed subcutaneously onto each side of the coronal sutures of 8-day-old *Twist-1*^del/+^ mice up to 20 days of age, in accordance with the SAHMRI Animal Ethics Approval (Ethics# SAM347). The calvaria of treated mice fixed in 10% formalin was analyzed using Masson’s trichrome staining. Mineralized calvarial bone formation relative to the length of bone analyzed was quantitated using OsteoMeasure software.

### Immunohistochemical analysis

Calvaria was isolated from 10-day-old Twist-1^del/+^ and wildtype mice and then fixed in 10% formalin for 24 h, decalcified with 14% EDTA (pH 7.2) overnight, and embedded in paraffin. The samples were cut transversely with thickness of 5 μm and processed as previously described [33]. The primary antibody used was an anti-mouse H3K27me3 rabbit polyclonal antibody (Cat# 07-449, Millipore, Bayswater, VIC, Australia). Rabbit IgG (Cat# I5006, Sigma–Aldrich) replaced the primary antibody as negative control, which showed no immunoreactivity. The percentage of H3K27me3-positive nuclei (brown) to total number of nuclei within the white box was quantitated using ImageJ software.

### Western blot analysis

Calvarial cells were cultured at 8 × 10^3^ cells/cm^2^ in T75 flask until confluent and then treated with GSK-J4 concentration range or 0.1% DMSO as vehicle control for 24 h. Histone extraction protocol was adapted from Abcam (Abcam, Melbourne, VIC, Australia). Briefly, 5 × 10^6^ cells were re-suspended in 1 ml of Triton Extraction Buffer (0.5% Triton X 100 (v/v), 2 mM phenylmethylsulfonyl fluoride (PMSF), 0.02% NaN_3_ (w/v)). The nuclei lysates were incubated on ice for 10 min with gentle stirring and centrifuged for 10 min at 6500 g at 4 °C. Histone acid extraction was performed using 0.2 M HCl at a density of 2 × 10^7^ nuclei/mL overnight at 4 °C. Histone protein was collected in the supernatant following centrifuge spin as described before and neutralized with 2 M NaOH at 1/10 of supernatant volume. Protein concentrations were measured using the Pierce Detergent Compatible Bradford Assay Kit (Cat# 1863028, Thermo Fisher Scientific). Western blot analysis was performed as described [50]. The membranes were blocked with 5% BSA blocking solution and probed overnight at 4 °C with an anti-mouse H3K27me3 rabbit polyclonal antibody (Cat# 07-449, Millipore Corporation, North Ryde, NSW, Australia) and a rabbit anti-H4 antibody (Cat#ab10158, Abcam). Following two washes with TBS/0.1% Tween 20, the blots were incubated for 1 h in room temperature with fluorescence secondary antibody (anti-rabbit 800 nm or 680 nm, Li-Cor Biosciences, VIC, Australia). Blots were washed two more times and then scanned on Odyssey CLX Near-Infrared Fluorescence Imaging System (Li-Cor Biosciences). Analysis and measurements were performed on Image Studio Lite software (Li-cor Biosciences).

### Chromatin immunoprecipitation (ChIP) analysis

Calvarial cells were seeded at density of 8 × 10^3^ cells/cm^2^ in T75 flasks. Once the cells were confluent, the cells were cultured in growth or osteogenic-inductive media in the presence of GSK-J4 at 1 μM or vehicle control (0.1% DMSO) for 24 h. ChIP protocol was adapted from Abcam. Chromatin was crosslinked with a final of 0.75% formaldehyde for 10 min at room temperature with gentle rocking. Glycine at a final concentration of 125 mM was added and incubated whilst shaking for 5 min. After two washes with PBS, adherent cells were detached using 1x trypsin, and the remaining cells were scraped. Cells were lysed with FA lysis buffer (50 mM HEPES KOH pH 7.5, 140 mM NaCl, 1 mM EDTA pH 8, 1% Triton X-100, 0.1% Sodium deoxycholate, 0.1% SDS, and protease inhibitors) at 400 ul per one million cells. DNA was sheared with a probe sonicator (Diagenode Bioruptor Inc., Denville, NJ, USA) on ice and then used for immunoprecipitation as previously described [53]. Primary antibodies that were used for immunoprecipitation were anti-mouse H3K27me3 rabbit polyclonal (1 mg/ml; Cat# 07-449, Millipore) and IgG rabbit polyclonal control (1 mg/ml Millipore). Transcription start site (TSS) primer sets (GeneWorks Pty Ltd) used in this study: mouse *Runx2* TSS (Fwd: 5′-AGGCCTTACCACAAGCCTTT-3′; Rev.: 5′-GTGGGACTGCCTACCACTGT-3′), mouse *Alkaline Phosphatase* TSS (Fwd: 5′-AGGGAAAGAGAGAGGCAAGG-3′, Rev.: 5′-TTCCTTACCTGCAGGCACTC-3′).

### Statistics

Experiments were performed in triplicates. Calculation of statistical significance was carried out using GraphPad PRISM 8 (GraphPad Software, La Jolla, CA, RRID: CR_002798, http://www.graphpad.com/). The software was also used for the generation of graphs which showed statistical differences (*) of *p* < 0.05 between samples.

## Results

### *Twist-1*^del/+^ calvarial cells exhibit increased expression and upregulated enzymatic activity of Kdm6a and Kdm6b

**Fig. 1:**
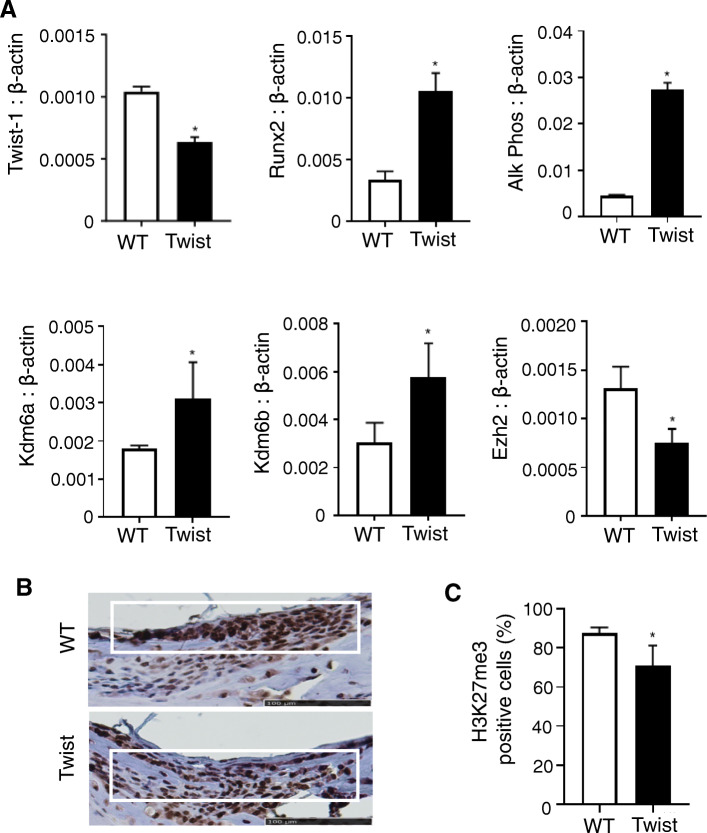
*Twist-1*^del/+^ calvarial cells exhibit differential expression of histone demethylases, Kdm6a and Kdm6b. **a** Gene expression levels of *Twist-1*, *Runx2, Alkaline Phosphatase (Alk Phos)*, *Kdm6a, Kdm6b,* and *Ezh2* in calvarial cells from wild-type (WT) and *Twist-1*^del/+^ (Twist) mice, cultured under osteogenic conditions were analyzed with real-time qPCR and normalized to *β-actin*. Data represent mean ± S.E., *p < 0.05, two-tailed, not-paired, non-parametric student’s *t* test, *n* = 3 WT, and *n* = 3 Twist mice. **b** Representative images of calvarial sections focusing on open coronal sutures (white box) of 8-day-old WT and Twist mice using an antibody specific to H3K27me3 (brown stain) counterstained with hematoxylin, scale bar = 100 μm. **c** Quantitative measurement of the percentage of H3K27me3-positive nuclei to total number of nuclei within the white box using ImageJ software. Data represent mean ± S. E, *p < 0.05, two-tailed, not-paired, non-parametric student’s *t* test, *n* = 3 WT, and *n* = 3 Twist mice)

Calvarial cells derived from 15-day-old *Twist-1*^del/+^ mice cultured under osteogenic inductive conditions were found to express reduced transcript levels of *Twist-1* and *Ezh2*, whereas gene expression levels of *Kdm6a*, *Kdm6b*, and the early (*Runx2*) and late (*alkaline phosphatase*) bone-associated markers were upregulated, compared to wild type calvarial cells (Fig. 1a). Immunohistochemical analysis demonstrated a decrease in H3K27me3-positive cells within the coronal sutures of day 8 (pre-fusion) *Twist-1*^del/+^ mice (Fig. 1b, c).

### Kdm6a and Kdm6b promote the osteogenic differentiation capacity of *Twist-1*^*del/+*^ calvarial cells

**Fig. 2:**
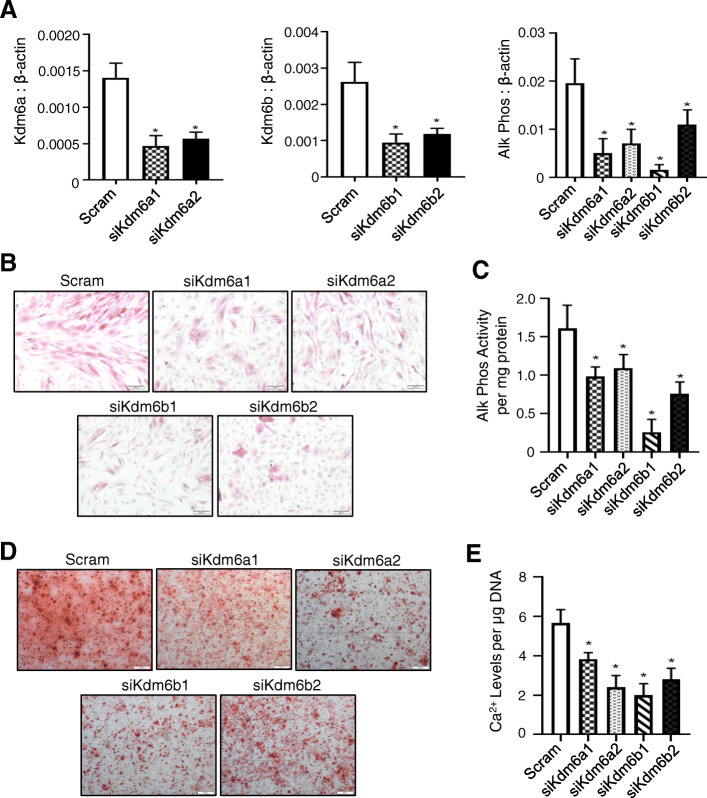
Kdm6a and Kdm6b promote osteogenic differentiation in *Twist-1*^del/+^ calvarial cells. **a** Real-time qPCR analysis of *Kdm6a*, *Kdm6b,* and *Alkaline Phosphatase* (Alk Phos) levels in *Twist-1*^del/+^ calvarial cells treated with siRNAs targeting either *Kdm6a* (siKdm6a1 or siKdm6a2) or *Kdm6b* (siKdm6b1 or siKdm6b2), compared to the siRNA scrambled control (Scram), under osteogenic inductive conditions. Data represent mean gene expression levels normalized to β-actin ± S.E. expression, *p < 0.05, two-tailed, not-paired, non-parametric student’s *t* test, *n* = 3 *Twist-1*^del/+^ mice. **b** Representative images of alkaline phosphatase staining of *Twist-1*^del/+^ calvaria cells treated with siRNA Scram control or *Kdm6A* and *Kdm6B*-specific siRNA, following 1 week of osteogenic induction, scale bar = 100 μm at ×50 magnification. **c** Quantitative analysis of alkaline phosphatase activity relative to total protein for *Twist-1*^del/+^ calvaria cells treated with Scram control and *Kdm6a* and *Kdm6b*-specific siRNA, following osteogenic induction. Data represent mean ± S.E., *p < 0.05, two-tailed, not-paired, non-parametric student’s *t* test, *n* = 4 *Twist-1*^del/+^ mice. **d** Representative images of Alizarin Red mineral staining of *Twist-1*^del/+^ calvaria cells treated with siRNA Scram control or *Kdm6A-* and *Kdm6B*-specific siRNA, following 2 weeks of osteogenic induction, scale bar = 100 μm at ×50 magnification. **e** Analysis of extracellular calcium levels relative to total DNA for *Twist-1*^del/+^ calvaria cells treated with siRNA Scram control or *Kdm6A-* and *Kdm6B*-specific siRNA, following 2 weeks of osteogenic induction. Data represent mean ± S.E*.,* *p < 0.05, two-tailed, not-paired, non-parametric student’s *t* test, *n* = 4 *Twist-1*^del/+^ mice

The role of Kdm6a and Kdm6b during osteogenic differentiation in calvarial cells from *Twist-1*^*del/+*^ mice was assessed using two specific siRNA molecules targeting either *Kdm6a* or *Kdm6b*. Reduced gene expression levels of *Kdm6a* or *Kdm6b* and the mature osteoblast marker, *Alkaline Phosphatase* was confirmed by qRT-PCR analysis following siRNA treatment (Fig. 2a). Furthermore, the data showed a decrease in alkaline phosphatase enzymatic activity in siRNA *Kdm6a* or *Kdm6b* transfected *Twist-1*^del/+^ calvarial cells under osteogenic conditions, compared with scrambled siRNA controls (Fig. 2b, c). Parallel studies found that the level of Alizarin red-positive mineralized deposits was significantly reduced in cultures of siRNA *Kdm6a* or *Kdm6b* knockdown *Twist-1*^del/+^ calvarial cells under osteogenic conditions, compared with scrambled siRNA-treated cells (Fig. 2d). This was confirmed by reduced amounts of extracellular calcium levels observed in replicate cultures of siRNA *Kdm6a*- or *Kdm6b*-treated *Twist-1*^del/+^ calvarial cells, compared to the scrambled siRNA controls (Fig. 2e).

### Kdm6a and Kdm6b inhibitor, GSK-J4, shows minimal toxicity in *Twist-1*^del/+^ calvarial cells

**Fig. 3:**
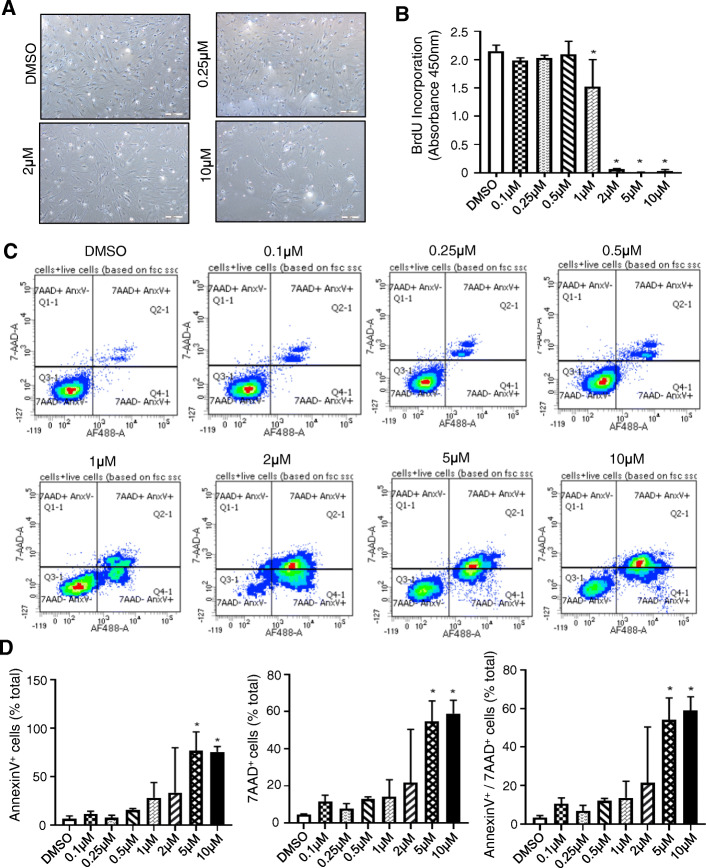
Effects of GSK-J4 on *Twist-1*^del/+^ viability and proliferation. **a** Representative cell densities of *Twist-1*^del/+^ calvarial cells are shown following treatment with low, medium, and high doses of GSK-J4 for 1 week, scale bar = 100 μm at ×50 magnification. **b** Proliferation rates were measured by BrdU incorporation for *Twist-1*^del/+^ calvarial cells following GSK-J4 treatment with a range of concentrations (0.1 μM–10 μM or 0.1% DMSO vehicle control) for 1 week. Data represent mean ± S.E., one-way ANOVA with Tukey’s multiple comparisons, *n* = 3 *Twist-1*^del/+^ mice. **c** Flow cytometric analysis of Annexin V/7AAD staining in *Twist-1*^del/+^ calvarial cells in the presence of GSK-J4 (0.1 μM–10 μM or 0.1% DMSO vehicle control) for 1 week. Representative histograms depicting early apoptotic cells (Annexin V^+^), necrotic cells (7AAD^+^), and late apoptotic cells (Annexin V^+^/7AAD^+^). **d** Quantitation of percentage of Annexin V/7AAD stained *Twist-1*^del/+^ calvarial cells by flow cytometric analysis in the presence of GSK-J4 (0.1 μM–10 μM or 0.1% DMSO vehicle control) for 1 week. Data represent mean ± S.E., *p < 0.05, one-way ANOVA with Tukey’s multiple comparisons, *n* = 3 *Twist-1*^del/+^ mice

*Twist-1*^del/+^ calvarial cells were cultured with increasing concentrations of GSK-J4 to assess potential cytotoxic effects. Observable differences in cell density occurred in the presence of GSK-J4 between 2 and 10 μM (Fig. 3a). Quantitative analysis found that the proliferation rate was not affected in the presence of 0.1–0.5 μM GSK-J4, but cell proliferation was significantly reduced between 1 and 10 μM GSK-J4, as assessed by BrdU incorporation (Fig. 3b). Flow cytometric analysis of *Twist-1*^del/+^ calvarial cells showed that the percentage of early apoptotic (Annexin V positive), necrotic (7AAD positive), and late-stage apoptotic (Annexin V + 7AAD-positive) cells significantly increased with GSK-J4 treatment at the higher doses of 5 μM and 10 μM (Fig. 3c, d). Therefore, concentrations higher than 2 μM were eliminated from further studies.

### Inhibition of Kdm6a and Kdm6b activity by GSK-J4 suppresses the osteogenic differentiation of *Twist-1*^*del/+*^ calvarial cells in vitro

**Fig. 4:**
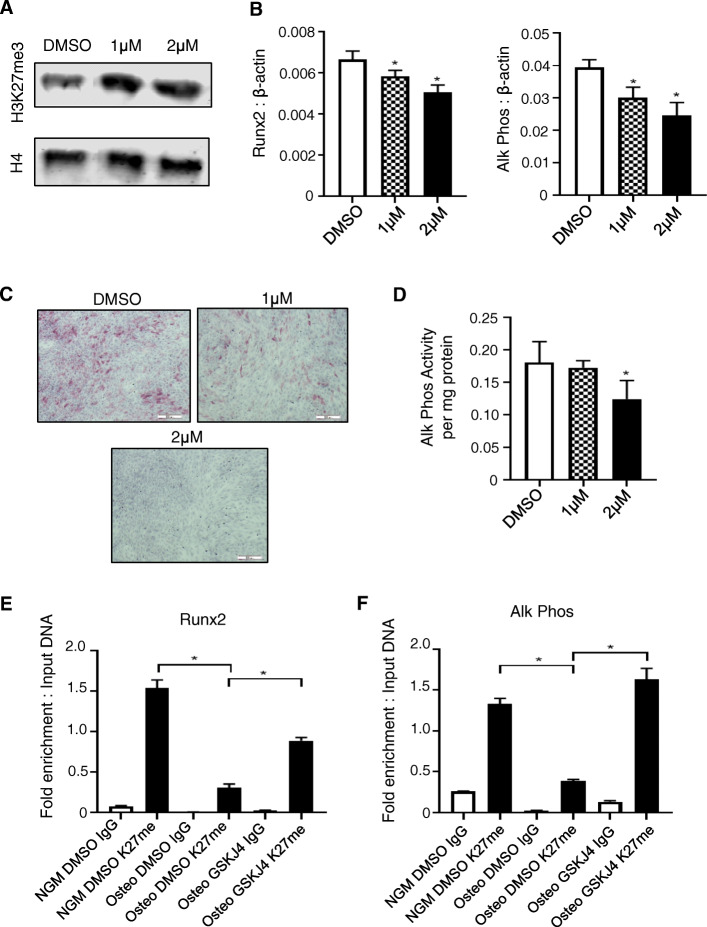
Inhibition of Kdm6a and Kdm6b activity suppresses osteogenic differentiation of *Twist-1*^del/+^ calvarial cells. **a** Western blot analysis of nuclear extracts isolated from *Twist-1*^del/+^ calvaria cells treated with GSK-J4 (1μM, 2 μM) or 0.1% DMSO vehicle control for 24 h to assess H3K27me3 levels relative to histone 4 (H4). **b** Real-time qPCR analysis of *Runx2* and *Alkaline Phosphatase* (Alk Phos) transcript levels in *Twist-1*^del/+^ calvaria cells under osteogenic inductions for 24 h. Data represent mean gene expression levels normalized to β-actin ± S.E. expression, *p < 0.05, two-tailed, not-paired, non-parametric student’s *t* test, *n* = 3 *Twist-1*^del/+^ mice. **c** Representative images of alkaline phosphatase staining and **d** quantitation of alkaline phosphatase activity relative to total protein for *Twist-1*^del/+^ calvaria cells treated with either 1 μM or 2 μM GSK-J4 or 0.1% DMSO, following 1 week of osteogenic induction. Data represent mean ± S.E., *p < 0.05, one-way ANOVA with Tukey’s multiple comparisons, *n* = 4 *Twist-1*^del/+^ mice. Chromatin immunoprecipitation (ChIP) analysis of H3K27me3 levels on the transcriptional start sites of **e** *Runx2* and **f** *Alk Phos* for *Twist-1*^del/+^ calvarial cells cultured under normal growth media (NGM) or osteogenic inductive conditions (Osteo) for 1 week in the presence of either GSK-J4 (1 μM) or 0.1% DMSO. ChIP was performed using either IgG control antibody (IgG) or H3K27me3-specific antibody (K27me). Enriched genomic DNA was used to amplify the transcription start site of target genes. Data represent mean fold enrichment relative to input DNA ± S.E., *p < 0.05, one-way ANOVA with Tukey’s multiple comparisons, *n* = 4 *Twist-1*^del/+^ mice

We next assessed whether inhibition of Kdm6a and Kdm6b activity could suppress the osteogenic differentiation capacity of *Twist-1*^del/+^ calvarial cells. Western blot analysis showed an increase in H3K27me3 levels in histone lysates extracted from *Twist-1*^del/+^ calvarial cells treated with 1 μM or 2 μM GSK-J4, compared to 0.1% DMSO (Fig. 4a), confirming the specificity of GSK-J4. Furthermore, *Twist-1*^del/+^ calvarial cells exhibited a reduction in *Runx2* and *Alkaline Phosphatase* gene expression levels in the presence of 1 or 2 μM of GSK-J4 compared to vehicle alone controls, when cultured under osteogenic inductive conditions (Fig. 4b). Supportive studies showed that alkaline phosphathase activity was significantly suppressed in *Twist-1*^del/+^ calvarial cells treated with 2 μM of GSK-J4, compared to vehicle controls (Fig. 4c, d). Chromatin collected from replicate experiments was used to analyze levels of the inhibitory mark, H3K27me3, present on the *Runx2* and *Alkaline Phosphatase* promoter transcription start sites (TSS), using ChIP analysis. The results demonstrated that H3K27me3 levels decreased dramatically on the *Runx2* and *Alkaline Phosphatase* TSS under osteogenic inductive conditions compared to normal growth conditions (Fig. 4e, f). However, treatment with GSK-J4 resulted in increased levels of H3K27me3 on the *Runx2* and *Alkaline Phosphatase* TSS, correlating with the suppression of these genes following GSK-J4 treatment (Fig. 4e, f). These findings suggested that the addition of GSK-J4 to Twist-1^del/+^ calvarial cell cultures increased the amount of H3K27me3 found on the promotors of osteogenic genes by inhibiting the activity of Kdm6a and Kdm6b during osteogenesis.

**Fig. 5:**
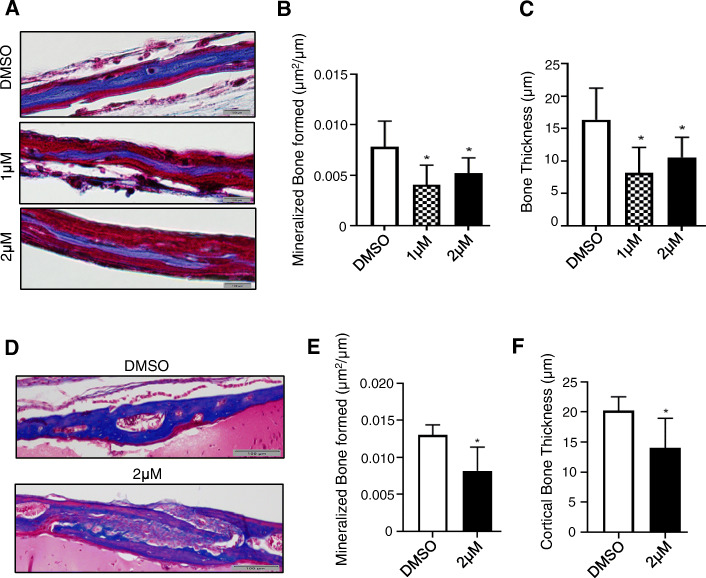
GSK-J4 treatment suppresses osteogenesis and prevents coronal suture fusion and bone formation of *Twist-1*^del/+^ mice. **a** Representative images of the stained *Twist-1*^del/+^ calvarial explants treated with BMP2 for 10 days in the presence of either 1 and 2 μM of GSK-J4 or 0.1% of DMSO vehicle control, then stained with Masson’s. The blue stain depicts the mineralized bone and the red stain depicts unmineralized osteoid, scale bar = 100 μm. Images captured at ×400 magnification. Histomorphometric analysis of **b** mineralized bone formed and **c** bone thickness of treated calvarial explants (*p < 0.05, one-way ANOVA with Tukey’s multiple comparisons, *n* = 5–7 *Twist-1*^del/+^ mice/treatment group). **d** Representative images of Masson’s trichome-stained coronal sutures of 20-day-old *Twist-1*^del/+^ mice following local implantation of CollaCote sponge carriers containing either 2 μM GSK-J4 or 0.1% DMSO at postnatal day 8, scale bar = 100 μm. Images captured at ×100 magnification. Histomorphometric analysis of **e** mineralized bone formed and **f** cortical bone thickness of locally treated coronal sutures (*p < 0.05, one-way ANOVA with Tukey’s multiple comparisons, *n* = 5–6 *Twist-1*^del/+^ mice/treatment group)

The effect of GSK-J4 on osteogenic differentiation was further examined using a murine calvarial organotypic explant model. Calvaria derived from *Twist-1*^del/+^ mice were cultured in media containing BMP2, to initiate bone formation, in the presence or absence of GSK-J4. The calvarial explants were then stained with Masson’s trichrome stain to identify newly mineralized bone (Fig. 5a). Histomorphometric analysis revealed a reduction in total bone formation and thickness in calvarial explants treated with 1 μm and 2 μM of GSK-J4, compared to 0.1% DMSO vehicle alone treated explants (Fig. 5b, c).

### GSK-J4 treatment prevents craniosynostosis in *Twist-1*^*del/+*^ mice

The ability of GSK-J4 treatment to prevent fusion of the coronal sutures in vivo was assessed using 3 mm^2^ Collacote™ sponges containing either DMSO alone or GSK-J4 placed subcutaneously on top of the coronal sutures in pre-fusion from 8-day-old *Twist-1*^*del/+*^ mice. Sections of coronal sutures derived from 20-day-old *Twist-1*^*del/+*^ mice were stained with Masson’s trichrome stain and examined by histomorphometric analysis. The data showed that 80% of *Twist-1*^*del/+*^ mice treated with 2 μM GSK-J4 exhibited open coronal sutures at postnatal day 20, whereas unilateral or bilateral coronal craniosynostosis was observed in 83% of the *Twist-1*^del/+^ mice treated with DMSO alone (Table 1 and Fig. 5d). Furthermore, there was a significant reduction in the total mineralized bone formed and bone thickness in the coronal sutures of GSK-J4-treated *Twist-1*^*del/+*^ mice compared to DMSO treated control mice (Fig. 5e, f). These findings demonstrate that local administration of GSK-J4 underneath the skull cap can prevent premature coronal suture fusion that occurs in *Twist-1*^del/+^ mice between postnatal days 9–20. Table 1 shows the number of *Twist-1*^del/+^ mice with open coronal sutures, unilateral or bilateral coronal craniosynostosis following treatment of either DMSO (0.1%) or 2 μM GSK-J4 (*n* = 5–6 *Twist-1*^del/+^ mice/treatment group).

**Table 1:** GSK-J4 treatment prevents unilateral and bilateral craniosynostosis in *Twist-1*^del/+^ mice

	DMSO control	2 μM GSK-J4
Open coronal sutures	1	4
Unilateral coronal craniosynostosis	2	1
Bilateral coronal craniosynostosis	3	0

## Discussion

In this study, we demonstrate for the first time that calvarial cells extracted from *Twist-1*^*del/+*^ mice express elevated levels of the H3K27me3 demethylases, *Kdm6a* and *Kdm6b*, whereas the gene expression levels of the counter histone methyltransferase, *Ezh2*, was reduced, compared to cells derived from littermate wildtype mice. This correlated to a reduction in the amount of H3K27me3 within the coronal sutures of *Twist-1*^del/+^ mice. These findings suggest that the altered balance in epigenetic enzymes that deposit or remove H3K27me3 are pivotal in driving the craniosynostosis phenotype. Both histone demethylases have been previously reported to promote osteogenic differentiation by removing the repressive mark, H3K27me3, on the promoter of osteogenic-promoting genes [41]. Similarly, we found that inhibition of Kdm6A and Kdm6b activity in *Twist-1*^del/+^ calvarial cells reduced gene transcript levels of *Runx2* and *Alkaline Phosphatase*, following treatment with GSK-J4, correlating to lower levels of H3K27me3 on the respective promoters. Previous studies have shown that TWIST-1 induces *EZH2* in cultured human mesenchymal stem cells increasing levels of *EZH2* and H3K27me3 along the Ink4A locus and bone gene promoters to increase proliferation but suppress osteogenesis, which was diminished in cranial bone cells derived from SCS patients [32, 33, 41]. These observations support our findings that *TWIST-1* mutations lead to aberrant *EZH2* and *KDM6A/B* expression levels, suggesting that a balance of histone demethylases and methyltransferase is essential in maintaining the correct fate determination of cranial MPC. The epigenetic dysregulation seen in the *Twist-1* haploinsuficient cells may therefore mediate the premature maturation of bone cells as a result of *Twist-1* mutation within the suture mesenchyme of the SCS mouse model. The aberrant osteogenesis in *Twist-1* mutant cells has been reported previously in other studies and further confirmed by the present study with the increased expression of *Runx2* and *Alkaline Phosphatase* when compared to wildtype cells [28, 33, 54]. Both of these osteogenic genes have been previously shown to be expressed within mouse calvarial cells at the osteogenic fronts and within the suture mesenchyme and thus have essential roles in the development of mouse calvaria [55, 56].

Functional studies using *Twist-1*^del/+^ calvarial cells determined that suppressing the expression of *Kdm6a* and *Kdm6b* led to the inhibition of early osteogenic differentiation shown in the reduced activity and expressions of *Alkaline Phosphatase*, and late osteogenic differentiation as seen in the reduced amount of mineral deposition and reduced calcium production. This is in agreement with previous studies reporting that *Kdm6b*-null mice display open calvarial sutures and less mineralized calvarial bones [46], whereas *Kdm6a*-null mice exhibit defects in neural crest formation [44]. Notably, the defects in *Kdm6a* knockout mice are more severe in female mice than in males [57, 58]. This suggested that Uty/Kdm6c, an enzymatically inactive paralog of Kdm6a located on the Y-chromosome, is able to compensate for the loss of Kdm6a in males [59, 60]. However, comparison between the sexes showed similar effects on osteogenesis following Kdm6a and Kdm6b inhibition (data not shown). This indicated that the enzymatic activity is essential in the regulation of H3K27me3 levels during calvarial osteogenic differentiation, and thus, Kdm6c activity was not able to compensate during Kdm6a and Kdm6b inhibition in this instance. Collectively, our findings provided evidence that Kdm6a and Kdm6b are putative targets in treating craniosynostosis in *Twist-1*^del/+^ mutant mice and confirmed previous studies that deregulated epigenetic patterns play significant roles in the development of craniosynostosis [23, 24].

Our study further explored the utility of a chemical inhibitor, GSK-J4, in suppressing the osteogenic potential of *Twist-1*^del/+^ calvarial cells. This inhibitor was designed to target KDM6A and KDM6B enzymatic activities by competitively binding with their active sites, responsible for the interaction between the co-substrate, α-ketoglutarate, and a histone-3 peptide [61]. Our study showed that the treatment of GSK-J4 using concentration of 1 μM and 2 μM on *Twist-1*^del/+^ calvarial cells resulted in a reduction in *Runx2* and *Alkaline Phosphatase* gene expression and activity. Notably, the dosage used had little or no effect in the cell viability rate of the *Twist-1*^del/+^ calvarial cells; however, higher doses significantly reduced the proliferation rate. This anti-proliferative effect of GSK-J4 has been previously described for embryonic bodies and tumor cells such as bone sarcoma [62], pediatric brain glioma [63], and acute lymphoblastic leukemia [64]. Whilst the reduction of proliferation was found to be caused by accumulation of cells at S-phase inhibiting cell cycle progression, our study showed that *Twist-1*^del/+^ calvarial cells undergo apoptosis in the presence of 5 μM GSK-J4.

Functional studies showed that GSK-J4 treatment reduced the development of the total bone on whole calvarial organotypic explant cultures derived from *Twist-1*^del/+^ mice. In a pre-clinical model of SCS, local administration of GSK-J4 to the calvaria of *Twist-1*^del/+^ mice prevented premature coronal suture fusion and reduced the amount of mineralized calvarial bone formation. The local delivery approach of GSK-J4 administration was performed because Kdm6a is ubiquitously expressed and both Kdm6a and Kdm6b were shown to be essential in the correct skeletal patterning, brain and heart development [43, 46, 57, 65, 66]. Collectively, the present study demonstrated that GSK-J4 treatment effectively suppressed osteogenic differentiation of *Twist-1*^del/+^ calvarial cells in both sexes and whole calvaria explants in vitro, and prevented coronal suture craniosynostosis of Twist-1^del/+^ mice in vivo by inhibiting the enzymatic activity of aberrant Kdm6a and Kdm6b levels and thus recovering the level of H3K27me3 marks on osteogenic genes.

Previous studies have employed GSK-J4 to understand the roles of the KDM6 subfamily members in regulating differentiation of embryonic stem cells and bone marrow-derived mesenchymal stem cells [65]. Moreover, GSK-J4 has been highly utilized in the studies of novel therapeutic strategies against various types of diseases, including acute lymphoblastic leukemia [64], myeloid leukemia [67], osteoarthritis [65] and breast [68], prostate [69], ovarian cancers [70], and brainstem glioma [63]. Of note, the later study also showed that normal brain cells from healthy children are unaffected by GSK-J4 treatment. This implies that the use of localized GSK-J4 treatment to reverse craniosynostosis may have little or no adverse impact on brain development in children with SCS. However, further studies are required to perform pathological assessments of any potential GSK-J4 toxicity issues for various tissues and organs, as well as cognitive evaluations, using *Twist-1*^del/+^ mice in the absence of pre-clinical large animal models of SCS.

Currently, the main treatment for craniosynostosis involves an open calvaria remodeling surgery. This type of surgery might lead to serious complications such as cerebral contusions, cerebrospinal fluid leaks, hematomas, infections, and wound breakdowns [17, 18]. Additionally, in severe cases of craniosynostosis, there is often a need for a follow-up treatment with repeated surgery procedures and substantial hospitalization, as there is the possibility that sutures might fuse before the cranium has had the opportunity to expand appropriately to accommodate for the growing brain [71]. Despite the negative impacts on a patient’s wellbeing and health providers, therapies which do not involve invasive surgery have yet to be developed.

## Conclusions

In this study, we demonstrate for the first time that the histone demethylases, Kdm6a, and Kdm6b have the potential to be used as novel therapeutic targets for the prevention of craniosynostosis in an SCS mouse model, where the *Twist-1* gene is mutated. Therefore, this study could potentially minimize the need for invasive approaches by using a local drug therapy such as GSK-J4 to slow down the rate of premature suture fusion in children with SCS, which is reversible following cessation of treatment.

## Data Availability

The datasets used and/or analyzed during the current study are available from the corresponding author on reasonable request.

## Abbreviations

MPC: Mesenchymal progenitor cells
SCS: Saethre-Chotzen syndrome
H3K27me3: Tri-methylation on the lysine-27 of the histone-3 tail
EZH2: Enhance of Zeste Homolog 2
KDM6A: Lysine demethylase 6A
KDM6B: Lysine demethylase 6B
αMEM: α-Modification of Eagle’s medium
DMSO: Dimethyl sulfoxide
BrdU: Bromodeoxyuridine
PCR: Polymerase chain reaction
PMSF: Phenylmethylsulfonyl fluoride
ChIP: Chromatin immunoprecipitation
